# Hsp60-Bearing Exosomes in *Helicobacter pylori*-Induced Gastric Tumorigenesis: A Pathomorphological and Therapeutical Overview

**DOI:** 10.3390/cells14211652

**Published:** 2025-10-22

**Authors:** Melania Ionelia Gratie, Olga Maria Manna, Salvatore Accomando, Giovanni Tomasello, Francesco Cappello, Alberto Fucarino

**Affiliations:** 1 Department of Biomedicine, Neurosciences and Advanced Diagnostics (BIND), Institute of Human Anatomy and Histology, University of Palermo, 90127 Palermo, Italy; melania.gratie@gmail.com (M.I.G.); giovanni.tomasello@unipa.it (G.T.); 2 Department of Health Promotion, Mother and Child Care, Internal Medicine and Medical Specialties “G. D’Alessandro”, University of Palermo, 90127 Palermo, Italy; olgamaria.manna@unipa.it (O.M.M.); salvatore.accomando@unipa.it (S.A.); 3 Department of Theoretical and Applied Sciences, eCampus University, 22060 Novedrate, Italy

**Keywords:** Helicobacter pylori, gastric mucosa, heat shock proteins, exosomes, tumorigenesis

## Abstract

**Highlights:**

**What are the main findings?**

- This review identifies Hsp60-bearing extracellular vesicles (EVs)—both bacterial (GroEL in *H. pylori* OMVs) and host-derived (human exosomes)—as central mediators of the infection–inflammation–cancer axis in gastric mucosa.
- It also introduces the novel concept of the *muco-microbiotic layer* as a fifth, functionally distinct layer of the gastric wall, serving as the key site for vesicle-mediated host–microbe interactions.

**What is the implication of the main finding?**

- Recognizing Hsp60-bearing EVs as active pathogenic and immunomodulatory agents opens new diagnostic and therapeutic avenues, including EV-based biomarkers and Hsp60-targeted interventions.
- The proposed framework not only redefines gastric wall morphology but also provides a new conceptual and experimental basis for studying early events in *H. pylori*-associated gastric carcinogenesis.

**Abstract:**

Chronic infection with *Helicobacter pylori* is the leading environmental cause of gastric carcinogenesis, yet the molecular pathways remain incompletely defined. This review links *H. pylori*-derived outer membrane vesicles (OMVs) and host epithelial exosomes through their shared cargo of heat shock protein 60 (GroEL/Hsp60). We proposed the concept of the “muco-microbiotic layer” as a fifth, functionally distinct layer of the gastric wall, where bacterial and host extracellular vesicles (EVs) interact within the mucus–microbiota interface. In this compartment, OMVs carrying bacterial GroEL and exosomes containing human Hsp60 engage in bidirectional communication that may promote chronic inflammation and epithelial transformation, with putative participation of molecular mimicry. The high structural homology between microbial and human Hsp60 enables repeated immune exposure to trigger cross-reactive responses—potentially leading to autoimmune-driven tissue damage, immune tolerance, and immune evasion in pre-neoplastic lesions. This vesicular crosstalk aligns with the evolution from non-atrophic gastritis to atrophy, from intestinal metaplasia to dysplasia, and lastly adenocarcinoma. Therapeutically, targeting EV-mediated Hsp60/GroEL signaling might offer promising strategies: EV-based biomarkers for early detection, monoclonal antibodies against extracellular Hsp60/GroEL, modulation of vesicle release, and probiotic-derived nanovesicles to restore mucosal balance. Hence, recognizing the muco-microbiotic layer and its vesicle-mediated signaling provides a new framework for understanding the infection–inflammation–cancer axis and for developing diagnostic and therapeutic approaches in *H. pylori*-associated gastric cancer.

## 1. Introduction

Gastric cancer continues to represent a major global health burden, ranking among the top causes of cancer-related deaths worldwide [1]. A substantial body of epidemiological, clinical, and experimental evidence has firmly established *Helicobacter pylori* (*H. pylori*) infection as the primary environmental factor contributing to gastric carcinogenesis [2]. According to the Maastricht V/Florence Consensus Report, approximately 40–60% of the world’s population is currently infected with *H. pylori* or has been infected in the past. With reference to the population affected by dyspepsia, the prevalence of serious lesions in the upper gastrointestinal tract among those under 50 years old (ages 45–55) is notably low, varying by geography. Furthermore, a global meta-analysis revealed that the prevalence of gastric cancer is only 0.4%, and it is even lower in individuals in fewer than 45 regions [3]. While primary infection in adults or reinfection after successful eradication can occur, these cases are relatively uncommon, with an annual incidence rate of 0.3–0.7% in developed countries and 6–14% in developing countries [4]. Although it is estimated that half of the world’s population is infected with *H. pylori*, there are significant variations in infection rates both between and within countries. The prevalence can differ even within a single city and among subgroups, such as urban versus rural populations. Epidemiological data indicate that the primary determinant of infection prevalence is socioeconomic status during childhood, which is influenced by factors such as hygiene standards, sanitation, and population density. Consequently, the prevalence of infection is generally higher in developing countries.

Interestingly, some countries have observed a significant decline in infection prevalence over time, particularly among children and young adults. This trend is particularly pronounced in developed countries and those that have experienced rapid economic growth, leading to improved socioeconomic conditions. As a result, a gradual decline in the rates of peptic ulcer and gastric cancer is anticipated, as these conditions generally reflect the prevalence of *H. pylori* in the population. Indeed, for decades, the prevalence of peptic ulcer disease and gastric cancer has been decreasing overall in developed countries [5].

*H. pylori* is a microaerophilic, Gram-negative bacterium that can colonize the adverse niche of the human stomach and establish a long-term chronic infection lasting decades. The typical progression from asymptomatic gastritis to atrophic gastritis, intestinal metaplasia, dysplasia, and eventually invasive carcinoma exemplifies the multistep model of inflammation-driven tumorigenesis [6]. However, the mechanisms by which *H. pylori* drive the transformation of gastric epithelium are multifactorial and only partially elucidated.

Among the many virulence strategies adopted by *H. pylori*, increasing attention has been directed toward the bacterium’s capacity to engage in molecular dialog with host cells via extracellular vesicles (EVs) [7]. These include both *H. pylori*-derived outer membrane vesicles (OMVs) and host-derived exosomes, which are both nanosized lipid bilayer-enclosed structures involved in intercellular communication/crosstalk. These vesicles shuttle a broad range of bioactive molecules, including proteins, nucleic acids, lipids, and metabolites. Of particular interest are the heat shock proteins (HSPs), especially Hsp60 (GroEL in bacteria), which are frequently enriched in both bacterial OMVs and host exosomes [8]. HSPs are highly conserved molecular chaperones and are traditionally known for their intracellular roles in proteostasis and stress adaptation. However, when localized at the cell surface or released via vesicles, they acquire immunomodulatory functions and are increasingly recognized as damage-associated molecular patterns (DAMPs) [9].

Concurrently, epithelial cells subjected to prolonged infection, oxidative stress, and inflammation may undergo phenotypic changes that include the overexpression of Hsp60 at the plasma membrane. This surface-expressed Hsp60 can be internalized into multivesicular bodies (MVBs) through lipid raft-mediated endocytosis, ultimately being secreted via exosomes [10]. This process establishes a potentially pathogenic feedback loop: the release of Hsp60-bearing exosomes from dysplastic or pre-malignant epithelial cells may reinforce chronic inflammation and immune activation, or paradoxically, drive immune tolerance and tumor immune evasion [10]. Notably, the dual nature of extracellular Hsp60—as both a pro-inflammatory and a tolerogenic signal—makes it a compelling, yet enigmatic player in the gastric tumor microenvironment.

Importantly, the pathomorphological consequences of this vesicular exchange are beginning to be understood. EV-associated HSPs could shape the gastric epithelial landscape by modulating cell proliferation, apoptosis resistance, epithelial–mesenchymal transition (EMT), and immune cell recruitment. These effects, when sustained over time, can contribute to a microenvironment conducive to malignant transformation [11]. Furthermore, the presence of Hsp60 on the surface of neoplastic cells may render them susceptible to autoimmune targeting—a process that could either aid in immune surveillance or, under specific conditions, lead to immune escape through exhaustion or polarization of immune effectors [10].

**Figure 1:**
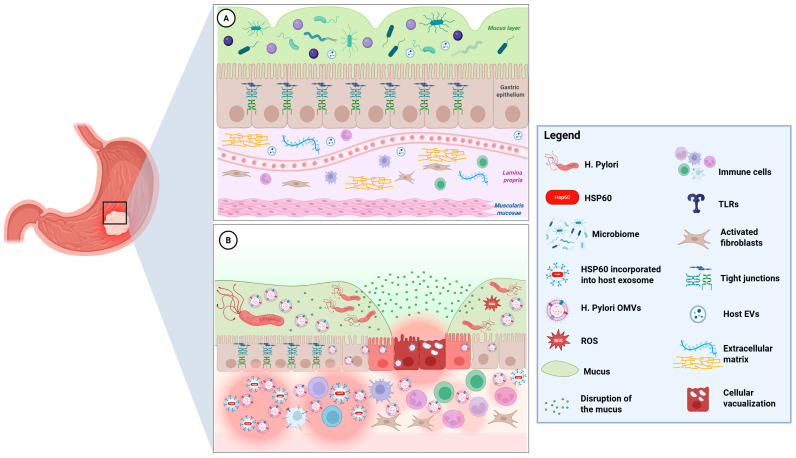
Schematic representation of the gastric mucosa under physiological conditions (**A**) and following infection with *H. pylori* (**B**). In (**A**), above the epithelium, the muco-microbiotic layer is depicted (in green) and is made of the mucus layer (produced by epithelial cells), the normal microbiota, and the nanovesicles produced by human cells and bacteria. See Paragraph 2 for more information. In (**B**), after *H. pylori* infection, the release of *H. pylori* OMVs causes numerous biological effects on host cells, leading to vacuolization, muco-microbiotic, and tight junctions between cells disruption, inflammation, and apoptosis. Oxidative, inflammatory, and bacterial stress induce the upregulation of Hsp60 in gastric epithelial cells. Notably, the crosstalk between *H. pylori* OMVs (containing also GroEL, the bacterial homolog of Hsp60, not showed) and host Hsp60-containing exosomes promotes the possibility of immune recognition via molecular mimicry and T cell or antibody autoimmune reactions against gastric cells, which can either promote chronic inflammation or lead to tissue damage and carcinogenesis. Further details about these putative pathogenetic mechanisms are explained in the next paragraphs (Image created with BioRender, (2025) https://BioRender.com/unhdnjo, Toronto, ON, Canada).

In this review, we aim to propose a novel interpretative framework that connects *H. pylori*-derived OMVs and host-derived exosomes through their shared cargo of Hsp60/GroEL. We explore the molecular and morphological implications of this vesicular crosstalk in the context of *H. pylori*-induced gastric tumorigenesis, with particular emphasis on the dual role of Hsp60 as a pro-tumorigenic mediator and potential autoantigen. Special attention is paid to the structural organization of the muco-microbiotic layer, the pathways of vesicle trafficking, and the emerging concept of EV-based molecular mimicry (Figure 1). By integrating insights from microbiology, immunology, and pathomorphology, we aim to elucidate how extracellular Hsp60 contributes to the transition from infection to malignancy, and to identify potential targets for therapeutic intervention and immune modulation in *H. pylori*-associated gastric diseases.

## 2. The Muco-Microbiotic Layer: A Novel Morpho-Functional Framework

Recent advances in gastrointestinal morphology and mucosal immunology have prompted us to introduce a reconceptualization of the gastric wall. Traditionally described as being composed of four concentric layers—mucosa, submucosa, muscularis propria, and serosa—both the stomach and the bowel wall description should include a fifth, functionally autonomous layer: the muco-microbiotic layer [12]. This layer constitutes, also in the stomach, the innermost component of the wall, lying directly between the gastric lumen and the epithelial surface of the *tonaca mucosa*, and represents a critical zone for host–environment interaction [13].

Although, at present, the muco-microbiotic layer is mainly a functional and hypothetical framework derived from our observations, its direct visualization can be attempted with mucin-preserving approaches. For instance, gastric biopsies can be rapidly frozen and sectioned with a cryostat to retain the mucus and the other components, thus allowing their microscopic evaluation by routinary staining (i.e., hematoxylin/eosin). Other complementary techniques, such as histochemistry/immunohistochemistry on frozen sections, cryo-electron microscopy, or even in vivo imaging modalities, may also provide valuable information for future confirmation and characterization of this layer.

This layer exists only in vivo and has likely not been described previously because it disintegrates during routine tissue processing for microscopic observation. This is because the most commonly used techniques involve alcohol-based dehydration, and alcohol dissolves the mucus, causing this layer to disappear. Nevertheless, recognizing the existence of this layer, as well as its cytological and molecular characterization, is crucial for a better understanding of the pathophysiological events affecting the inner part of the wall of these organs of the alimentary canal.

The muco-microbiotic layer of the stomach is composed of three tightly interconnected elements: (1) the mucus, secreted by surface and neck mucous cells, which forms a viscoelastic gel rich in glycoproteins (primarily MUC5AC and MUC6); (2) the microbiota, which includes both commensal and pathogenic organisms, among which *H. pylori* can be prominent in some pathological conditions; (3) extracellular vesicles (EVs), including OMVs of bacterial origin and exosomes released mainly by gastric epithelial cells.

Functionally, this layer acts as a bioactive interface: it not only protects the *tonaca mucosa* from acid and enzymes but also mediates molecular crosstalk between microbes and host tissues. The mucus provides a scaffold for microbial colonization and defines a niche that is biochemically distinct from the lumen, more stable in pH, and enriched with host-derived factors. In the context of *H. pylori* infection, this balance is disrupted [7] (Figure 1). The bacterium actively modifies the mucus layer by altering mucin expression, neutralizing acidity through urease, and penetrating the mucus to adhere directly to epithelial cells.

One of the hallmarks of the muco-microbiotic layer during infection is probably the accumulation of EVs. Indeed, *H. pylori*-derived OMVs contain virulence factors such as CagA, VacA, urease, and GroEL (bacterial homolog of Hsp60), while epithelial exosomes—especially under stress—carry, among other, human Hsp60, Hsp70, regulatory RNAs, and cytokines [8]. These vesicles may serve not only as vehicles of molecular communication but also as agents of immune modulation and potential initiators of neoplastic transformation.

From a pathophysiological standpoint, in our opinion, the muco-microbiotic layer could be putatively regarded as one of the primary zones of interaction where microbial signals, host stress responses, and immune regulation converge. In other words, it is here that *H. pylori* establish persistent colonization, where host cells release stress-induced vesicles, and where the first steps of chronic inflammation and epithelial remodeling take place. Over time, these interactions might culminate in barrier dysfunction, epithelial dedifferentiation, and dysplasia. Further studies will address and (dis)prove this hypothesis.

In brief, the muco-microbiotic layer must be considered not merely as a surface covering but a structurally and functionally distinct layer of the gastric wall, with a key role in maintaining mucosal equilibrium and in initiating pathological processes. Its recognition as the fifth layer (the innermost) of the gastric wall reframes our understanding of gastric anatomy and histology and provides a crucial conceptual tool for investigating the early events in *H. pylori*-associated gastric carcinogenesis. In the next paragraphs, we will report some evidence in support of that.

## 3. *H. pylori* GroEL and Host Hsp60 in Vesicle-Mediated Pathogenicity

Among the multiple strategies employed by *H. pylori* to establish chronic infection and manipulate the host environment, the production of OMVs could be a key mechanism of virulence and immune evasion [14,15,16]. OMVs are nanoscale, bilayer vesicles (20–450 nm) naturally released by Gram-negative bacteria during growth, stress, or active infection. These vesicles contain a complex molecular cargo that reflects the composition of the outer membrane and periplasmic space of the bacterium, including lipopolysaccharides, peptidoglycan, phospholipids, proteins, DNA, and small RNAs [17].

In *H. pylori*, OMVs have been shown to carry a rich repertoire of virulence-associated molecules, including the cytotoxins CagA and VacA, urease subunits, adhesins (BabA, SabA), and several chaperones, notably GroEL, which is said to be the bacterial homolog of Hsp60 [18]. Once released into the muco-microbiotic layer, these OMVs can interact with host epithelial cells and immune components, either by receptor-mediated binding, endocytosis, or direct membrane fusion. While this concept remains primarily theoretical at present, there is emerging evidence suggesting that outer membrane vesicles (OMVs) have the capacity to fuse with the membranes of immune cells. Additionally, it is possible that a similar interaction occurs with epithelial cells [17,19]. This vesicle-mediated delivery system would allow *H. pylori* to extend its influence beyond direct bacterial–host cell contact, reaching the bloodstream of the *lamina propria*, and in turn distant targets, putatively exerting systemic effects.

Among the OMV cargo, the role of GroEL in the immunopathogenesis of *H. pylori* infection has not been gaining enough attention for what has already been demonstrated in other types of bacteria. Indeed, although classically considered an intracellular protein involved in protein folding and stress response, GroEL can localize to the bacterial surface and is actively packaged into OMVs [20,21]. Proteomic analyses have consistently shown GroEL to be a prominent protein associated with outer membrane vesicles (OMVs) in *Helicobacter pylori* and other Gram-negative bacteria. This supports the idea that OMVs are the primary method through which this chaperonin is exported into the extracellular environment [22]. Once delivered to host tissues, GroEL may be recognized primarily by Toll-like receptor 4 (TLR4) and, to a lesser extent, TLR2 on epithelial and immune cells [23,24]. This interaction triggers NF-κB- and MAPK-signaling pathways, leading to the transcription of pro-inflammatory cytokines (e.g., IL-8 and TNF-α), which may contribute to neutrophil recruitment and chronic mucosal inflammation.

Notably, GroEL shares a high degree of structural conservation with human Hsp60, raising the possibility of molecular mimicry and cross-reactive immune responses [25]. Repeated exposure to GroEL-containing OMVs may prime T and B cells against epitopes that are also present on human Hsp60, particularly when the latter is mislocalized to the cell surface or secreted in exosomes during cellular stress. This could establish a chronic inflammatory loop, whereby immune recognition of bacterial GroEL may fuel a secondary autoimmune reaction against host tissues.

OMVs also play a role in modulating epithelial cell function and transformation. Delivery of CagA and VacA through OMVs can induce cytoskeletal rearrangements, tight junction disruption, vacuolization, and mitochondrial dysfunction [26,27]. In turn, these events could also promote increased proliferation and resistance to apoptosis—hallmarks of early neoplastic transformation, although these mechanisms remain to be fully clarified. In light of this evidence, understanding how *H. pylori* GroEL-bearing OMVs could shape the mucosal microenvironment may be useful for further clarifying the early steps of *H. pylori*-induced carcinogenesis and for identifying novel targets for immunomodulatory therapies.

Collectively, these findings position *H. pylori* OMVs not only as passive carriers of virulence factors but as active effectors of gastric epithelial remodeling. By delivering GroEL/Hsp60 and other stress proteins within the muco-microbiotic layer, OMVs can sustain NF-κB and MAPK activation, induce epithelial–mesenchymal transition-like changes, and promote resistance to apoptosis [14,15,16,17,18,26,27]. This vesicle-mediated delivery system thus amplifies chronic inflammation and creates a permissive microenvironment for neoplastic transformation, complementing the effects of host-derived Hsp60-bearing exosomes described in the following section.

The main pathogenic mechanisms through which *H. pylori* use OMVs, virulence factors, and host-derived exosomal proteins to cause chronic inflammation, immune modulation, and gastric carcinogenesis are described in this section and in the previous ones are summarized in Table 1.

## 4. Gastric Epithelial Cell Stress and Exosomal Hsp60

In the setting of chronic *H. pylori* infection, gastric epithelial cells are persistently exposed to oxidative stress, inflammatory mediators, and direct bacterial toxicity. These conditions activate a broad array of cellular stress responses, among which the heat shock response is particularly prominent. One of the central actors in this system is Hsp60, which is a mitochondrial chaperone that under stress can redistribute to extra-mitochondrial compartments, including the cytosol, the plasma membrane, and even the extracellular space, primarily via exosome secretion.

Under physiological conditions, Hsp60 is confined to the mitochondrial matrix, where it assists in protein folding and prevents misfolding-induced apoptosis [28]. However, during stress—especially oxidative, endoplasmic reticulum, or bacterial-induced stress—Hsp60 expression is upregulated, and the protein can translocate to the plasma membrane [10]. This membrane localization is a prerequisite for its packaging into MVBs, which subsequently fuse with the plasma membrane and release their content as exosomes. The incorporation of Hsp60 into exosomes is thought to occur via lipid raft-mediated internalization, which is a process enhanced by surface exposure of Hsp60 on the apical membrane of epithelial cells undergoing phenotypic transformation [10].

The presence of Hsp60 in epithelial-derived exosomes may have several biological implications. First, it can reinforce the notion that stressed or dysplastic epithelial cells are not passive victims of inflammation, but active participants in shaping the immune microenvironment. Exosomal Hsp60 can act as an alarmin [29], putatively signaling tissue danger and promoting the activation of innate immune receptors such as TLR4 on macrophages, dendritic cells, and adjacent epithelial cells [30]. This can lead to a paracrine amplification of inflammation, characterized by increased secretion of IL-6, IL-8, and TNF-α, thereby sustaining the chronic inflammatory milieu typical of *H. pylori*-infected mucosa.

Second, and perhaps more significantly, the release of Hsp60 via exosomes from pre-neoplastic or dysplastic cells introduces the possibility of immune recognition via molecular mimicry [34] (Table 1). Given the structural homology between bacterial GroEL and human Hsp60, T cells or antibodies initially primed against *H. pylori* OMV-associated GroEL may cross-react with epithelial Hsp60 displayed or secreted in the context of dysplasia. This autoimmune cross-recognition could target transformed cells for destruction—a mechanism resembling tumor immune surveillance [25]—or alternatively contribute to bystander tissue damage that accelerates carcinogenesis.

However, since pre-malignant and malignant cells exosomes are particularly enriched in Hsp60 [31], this supports the idea that immune recognition of these vesicles might serve as an early warning signal of malignant progression. Hence, in some contexts, persistent exposure to extracellular Hsp60 may lead to immune tolerance, the expansion of regulatory T cells, or functional exhaustion of cytotoxic lymphocytes—ultimately favoring immune escape and tumor development.

In addition to its immune-modulatory properties, exosomal Hsp60 may influence cellular behavior in an autocrine or paracrine fashion. Studies in other epithelial systems have shown that extracellular Hsp60 can promote cell migration, resistance to apoptosis, and even angiogenesis, though these functions are likely context/microenvironment-dependent and—to the best of our knowledge—remain underexplored in the gastric setting [31,32,38].

## 5. Vesicular Crosstalk and Molecular Mimicry

While the vesicle-mediated molecular mimicry between bacterial GroEL and human Hsp60 appears conceptually plausible and is supported by our previous observations, it remains at present a working model requiring further experimental validation.

In particular, the persistent colonization of the gastric mucosa by *H. pylori* establishes a complex and prolonged interaction between the bacterium and the host. At the heart of this interaction is likely also a bidirectional exchange of EVs. For instance, *H. pylori* releases OMVs laden with virulence factors, while the host epithelium responds by secreting exosomes enriched in stress-related molecules [7]. This vesicular crosstalk, occurring within the muco-microbiotic layer, may play a role in shaping the local microenvironment, modulating immune responses, and also putatively driving the transition from chronic inflammation to neoplastic transformation.

One of the most compelling aspects of this vesicle-mediated communication is the shared presence of GroEL and Hsp60, respectively, in OMVs and host exosomes [8]. These evolutionary homolog proteins display a high degree of structural and epitope similarity, which underlies a potential phenomenon of molecular mimicry [25]. As previously said, molecular mimicry occurs when immune responses initially directed against a microbial antigen cross-react with structurally similar host molecules, potentially leading to autoimmune reactions and/or chronic tissue injury. It has been proposed that human HSP60 may also function as a single ring and form symmetrical, football-shaped intermediates, much like GroEL. Despite its high sequence similarity to GroEL, human HSP60 exhibits some distinctive features in its oligomer organization. In conclusion, it can be suggested that different functional mechanisms, involving various types of oligomers, may be observed with HSP60 single rings [39]. It is likely that the chaperonin will follow a specific pathway based on the type of substrate and the micro-environmental conditions, such as those present during physiological and stress situation [40].

In the context of *H. pylori* infection, repeated exposure to GroEL-bearing OMVs may prime antigen-presenting cells (such as dendritic cells and macrophages) to process and present GroEL peptides on MHC molecules. Because of the high epitope similarity between GroEL and human Hsp60, these cells may subsequently display cross-reactive Hsp60-derived peptides from stressed epithelial cells in the same immunological context [25,34]. This scenario can polarize T cell responses along two opposite pathways. On one hand, pro-inflammatory Th1/Th17 responses and cytotoxic CD8^+^ T cells may be activated, producing cytokines such as IFN-γ, IL-17, or TNF-α, and mediating autoimmune-like tissue injury and epithelial destruction [40,41,42]. On the other hand, chronic and high-dose exposure to extracellular Hsp60 can favor tolerogenic dendritic cell phenotypes and expansion of regulatory T cells, with increased IL-10 and TGF-β production, leading to local immune suppression and the escape of dysplastic or neoplastic cells [41,42]. The balance between these effector and regulatory arms, together with the duration and intensity of antigen exposure, may ultimately determine whether molecular mimicry translates into protective immune surveillance or into immune tolerance and tumor progression [23,34,40,41,42].

Moreover, the simultaneous presence of bacterial- and host-derived EVs within the muco-microbiotic layer enhances the spatial and temporal proximity of these antigens, increasing the likelihood of antigenic overlap and cross-presentation by local antigen-presenting cells. Dendritic cells or macrophages encountering both OMVs and epithelial exosomes may process and present peptides from both sources in the same MHC context [35,36], blurring the immunological distinction between “self” and “non-self.” This antigenic convergence may favor the development of hybrid immune repertoires, with unpredictable consequences for immune tolerance and tissue integrity.

Indeed, this mimicry-driven mechanism does not uniformly lead to pathology. In some individuals, it might contribute to protective immunity, aiding in the elimination of tumoral and/or pretumoral cells [37]. In others, depending on genetic background, cytokine milieu, and microbiota composition, it might result in immune dysregulation [43], fostering either chronic inflammation or immune evasion. The final outcome may depend on a delicate balance between pro-inflammatory and regulatory pathways, as well as the duration and intensity of antigen exposure [34].

Adding complexity to this picture, exosomal Hsp60 may also act as a regulatory signal: in certain contexts, it has been shown to promote tolerogenic responses, including regulatory T cell expansion, monocyte deactivation, or NK cell suppression [40,41]. Thus, chronic exposure to extracellular Hsp60—whether bacterial- or host-derived—may paradoxically shift the immune response from activation to exhaustion or anergy, creating a permissive environment for tumor development. This dual role—danger signal vs. immune silencer—positions Hsp60 as a paradigmatic example of context-dependent immunomodulation [42].

Altogether, the interplay between GroEL-containing OMVs and Hsp60-enriched exosomes may create a molecular network of signals that extend beyond direct bacterial infection. It influences epithelial plasticity, immune cell polarization, and tissue homeostasis, possibly culminating in either immune-mediated tissue damage or immune escape mechanisms. In this light, the muco-microbiotic layer is not only a physical frontier but also an immunological interface where self and non-self antigens converge and interact via shared vesicular pathways.

Understanding this vesicle-driven molecular mimicry may open new avenues for research and clinical translation. Targeting vesicular release pathways, modulating immune recognition of extracellular Hsp60, or disrupting antigen presentation mechanisms can offer novel strategies to interrupt the infection–inflammation–cancer axis in *H. pylori*-associated disease. All these mechanisms are schematically summarized in Table 1, which highlights how vesicle-mediated GroEL/Hsp60 mimicry may drive either tissue injury or immune tolerance.

## 6. Pathomorphological and Immunological Implications of EV Crosstalks

It is intriguing to suppose that the crosstalk between *H. pylori* and the gastric mucosa mediated by extracellular vesicles bearing Hsp60 may culminate in a series of changes that could be either morphologically detectable or immunologically significant. These changes would not occur in isolation but as part of a progressive, multistep process of mucosal remodeling, immune polarization, and eventually, epithelial transformation.

Indeed, from a pathomorphological perspective, chronic *H. pylori* infection is classically associated with non-atrophic gastritis, which can evolve into atrophic gastritis, intestinal metaplasia, dysplasia, and ultimately adenocarcinoma [44]. Within this spectrum, early lesions already show signs of epithelial stress and vesicular activity. Morphological and biochemical analyses have demonstrated the exosomal release in inflamed gastric epithelium [45]. In parallel, *H. pylori* infection induces changes in gastric mucosa, both in epithelial and lamina propria layers, with several morpho-functional changes which reflect either a state of ongoing repair or a deregulated proliferation, the latter being a condition known to favor carcinogenesis [46].

In the infected mucosa, Hsp60 expression becomes abnormally upregulated, not only intracellularly but also on the plasma membrane and within the exosomal cargo, alone or associated with other molecules [47]. This aberrant localization is significant, as it provides the conditions for immune detection of epithelial cells as “altered” or “non-self.” Simultaneously, the persistent presence of GroEL in *H. pylori* OMVs maintains a low-grade inflammatory state [8], which is a histopathological frame characterized by lymphoplasmacytic infiltration and/or neutrophil accumulation and/or lymphoid follicle formation—a hallmark of MALT lymphomas development [48].

The immunological consequences of this environment are double-edged [8]. On one hand, innate immune activation via TLR2/TLR4 signaling promotes cytokine production (e.g., IL-8, IL-6, TNF-α), recruitment of neutrophils and macrophages, and antigen presentation. On the other hand, repeated exposure to Hsp60 in both microbial- and host-derived vesicles may drive adaptive immune exhaustion or immune deviation, favoring regulatory T cell expansion and local immune tolerance. This immunosuppressive shift may initially limit tissue damage but ultimately creates a permissive niche for neoplastic escape.

In the context of dysplasia and early carcinoma, epithelial cells not only overexpress Hsp60 but also exhibit increased vesicle trafficking, leading to a higher concentration of Hsp60-bearing exosomes in the extracellular space. These vesicles can further promote tumor progression through several mechanisms [8], including paracrine activation of neighboring cells (e.g., inducing EMT-like changes), autocrine survival signaling, and suppression of immune surveillance via modulation of NK cells or cytotoxic T lymphocytes.

Interestingly, ectopic expression of Hsp60 on dysplastic cell membranes has also been suggested as a target for immune recognition [38], potentially contributing to anti-tumor responses in early-stage lesions. However, as the tumor progresses, the same mechanisms might become co-opted by the neoplastic cells to actively suppress immune detection, through the release of tolerogenic exosomes.

Pathologically, these immuno-morphological changes are not yet integrated into standard diagnostic criteria, but they may represent emerging biomarkers for early detection and stratification of *H. pylori*-associated gastric lesions. Immunohistochemical detection of Hsp60 (both human and bacterial, i.e., GroEL) in biopsy samples, combined with vesicle-specific markers (e.g., CD63, CD81), might provide insight into the state of mucosal stress, immune activation, or evasion. Furthermore, the colocalization of Hsp60 with lipid raft domains in epithelial membranes might serve as a morpho-functional marker of exosomal trafficking potential. However, further investigation is needed to (dis)prove these hypotheses.

In any case, the pathomorphological and immunological consequences of Hsp60-bearing vesicle crosstalk in *H. pylori*-infected mucosa may be profound. They can span from subtle epithelial stress and immune activation in early gastritis, to immune suppression and neoplastic progression in advanced lesions. Recognizing and integrating these mechanisms into diagnostic and therapeutic frameworks will significantly advance the early detection, risk stratification, and immunomodulatory treatment of *H. pylori*-associated gastric cancer.

## 7. Diagnostic/Therapeutic Insights and Future Directions

Understanding the role of Hsp60-bearing EVs in *H. pylori*-associated gastric tumorigenesis may open novel avenues for both therapeutic intervention and disease monitoring. As EVs emerge not merely as byproducts of infection and cellular stress but as active mediators of inflammation, mimicry, and transformation, they become attractive targets and tools in the management of many diseases including gastric ones [33].

One of the most immediate translational implications concerns biomarker development [49]. The detection of Hsp60-positive exosomes and OMVs in gastric juice, blood, or tissue biopsies could offer a non-invasive or minimally invasive strategy to monitor mucosal stress, early epithelial dysplasia, or subclinical neoplastic transformation. EV-associated Hsp60 may also serve as a surrogate marker of mucosal immune dysregulation [50], helping to stratify patients at higher risk for progression to atrophic gastritis, intestinal metaplasia, or gastric cancer.

Moreover, therapeutic modulation of EV release or uptake represents a novel strategy [17,51]. Pharmacological inhibition of exosome biogenesis or release could potentially reduce the spread of pro-inflammatory or tolerogenic signals, limiting mucosal damage and immune evasion. Similarly, blockade of OMV–host cell interactions, using antibodies or small molecules targeting OMV-associated adhesins, TLR ligands, or GroEL itself, may attenuate the chronic inflammatory loop that underlies gastric pathology. Given their enrichment in GroEL and other immunomodulatory ligands, *H. pylori* OMVs themselves may represent attractive diagnostic targets or therapeutic vectors, complementing strategies aimed at extracellular Hsp60 [33].

A particularly compelling direction is the selective targeting of extracellular Hsp60. Because Hsp60 is normally confined to mitochondria, its extracellular or surface localization represents a stress- and transformation-specific signal, absent in most healthy tissues [32]. This unique feature may allow the design of Hsp60-directed therapies, such as monoclonal antibodies, peptide vaccines [52], or Hsp60-binding nanoparticles [50], capable of targeting pre-malignant or neoplastic cells. Such strategies might simultaneously eliminate dysplastic foci, reduce local inflammation, and promote immune surveillance—especially if combined with immune checkpoint blockade or T cell-based immunotherapies.

On the microbial side, efforts are underway to develop OMV-based vaccines against *H. pylori*, which exploit the immunogenicity of OMV-associated antigens without the risk of live infection [8]. However, the inclusion of GroEL or other conserved stress proteins in such formulations must be approached with caution, given their potential to elicit autoimmune reactions via molecular mimicry. Careful epitope mapping and selection of non-cross-reactive peptides will be essential to preserve vaccine efficacy while minimizing off-target effects.

Another emerging therapeutic field is the use of probiotic- or postbiotic-derived EVs as functional antagonists of *H. pylori* OMVs [7]. Certain Lactobacillus- and Bifidobacterium-derived vesicles exhibit anti-inflammatory, mucosal-barrier-enhancing, and even immune-regulatory properties. Administered orally or locally, these vesicles may help to restore balance to the muco-microbiotic layer, interfere with *H. pylori* adhesion and signaling, and modulate host immune responses toward resolution rather than persistence. Harnessing this potential may allow the development of biological EV-based adjuvants or therapeutics that integrate into *H. pylori* eradication regimens or preventive strategies in high-risk populations.

Finally, it is of worth to mention that the current management of *H. pylori* infection, relying primarily on antibiotic-based eradication therapies (bismuth quadruple therapy as the preferred first-line option) may not only successfully eradicate infection but also induce remission in early-stage gastric MALT lymphoma [53], highlighting the tight link between bacterial persistence and tumor development. However, for *H. pylori*-negative or refractory cases, radiotherapy, immunotherapy, or combination chemotherapy may be required, reflecting a stage- and response-adapted treatment approach [54].

All together, these findings would call in the future for a re-evaluation of histopathological assessment protocols. As discussed earlier, the muco-microbiotic layer is typically lost during standard tissue processing. In our opinion, incorporating mucin-preserving fixation techniques, vesicle-specific immunostaining, and Hsp60 immunolabeling in routine diagnostics may yield valuable information on early epithelial transformation and immune status, facilitating earlier and more precise clinical intervention.

In summary, the vesicle-mediated interplay between *H. pylori* and the host—centered on the presence of Hsp60—provides a rich therapeutic landscape spanning from biomarker discovery to immunotherapy, microbiome modulation, and vaccine design. Continued research into the molecular details of Hsp60 trafficking, recognition, and function within EVs will be very useful to translate these insights into effective and safe clinical applications. This paradigm shift—from viewing vesicles as epiphenomena to recognizing them as active drivers of disease and therapeutic targets—has the potential to transform our approach to gastric cancer prevention and management.

## 8. Conclusions

The path from *H. pylori* infection to gastric cancer is a paradigmatic example of inflammation-driven carcinogenesis, shaped by a continuous interplay between microbial virulence, host response, and environmental factors. Within this dynamic process, EVs—both bacterial and epithelial in origin—emerge not as passive markers of stress, but as active agents in the modulation of immune responses, epithelial transformation, and possibly immune surveillance.

Among the molecules shuttled by these vesicles, Hsp60 occupies a particular position. As a highly conserved chaperone, present in both *H. pylori* (i.e., GroEL) and human epithelial cells, Hsp60 may be viewed as a converging molecular signal that links infection, stress adaptation, and immune mimicry. OMVs containing GroEL activate innate immune receptors and sustain inflammation, while epithelial-derived exosomes carrying human Hsp60 may amplify stress signaling or become targets of cross-reactive immunity. This duality makes extracellular Hsp60 a central player in the mucosal environment, capable of driving either protective or pathogenic outcomes depending on context.

Crucially, these interactions take place within the muco-microbiotic layer, which we proposed to consider as the innermost layer of the gastric wall—i.e., the morpho-functional microenvironment integrating mucus, microbiota, and EVs. This layer should be recognized not only as a barrier but as a bioactive zone, where immune decisions are made, epithelial plasticity is modulated, and early neoplastic signals may arise.

Recognizing the pathomorphological and immunological implications of Hsp60-bearing vesicles opens the door to a range of diagnostic and therapeutic strategies. These include the development of EV-based biomarkers, selective targeting of extracellular Hsp60, modulation of vesicle biogenesis, and the use of probiotic-derived EVs to counteract pathogenic signaling. Moreover, rethinking histological and molecular approaches to include the muco-microbiotic layer in the gastrointestinal wall may help in molecular early detection of gastric neoplasia and provide deeper insights into epithelial–immune–microbial interactions.

In conclusion, the concept of Hsp60-bearing EVs playing a role in gastric tumorigenesis bridges microbial pathogenesis, cellular stress biology, and tumor immunology. Further investigation into this vesicle-based molecular dialog promises not only to clarify unresolved questions in *H. pylori*-associated disease, but also to putatively identify novel intervention points for gastric cancer prevention and therapy.

## Figures and Tables

**Table 1:** This table summarizes the major pathogenic mechanisms by which *H. pylori* employ OMVs, virulence factors, and host-derived exosomal proteins to drive chronic inflammation, immune modulation, and gastric carcinogenesis. Further details are explained in the text.

Pathogenic Mechanisms	Description	References
**Muco-Microbiotic** **Disruption**	*H. pylori* modify the mucus layer by altering mucin expression, neutralizing acidity with urease, and adhering to epithelial cells.	[7,12,13]
**OMVs-Mediated** **Pathogenesis**	OMVs play a critical role in the virulence and immune evasion of *Helicobacter pylori*. They carry various virulence factors, including CagA, VacA, urease subunits, and adhesins, as well as chaperones like GroEL, which is a bacterial equivalent of Hsp60. Furthermore, OMVs contribute to the modulation of epithelial cell function and transformation.	[14,15,16,17,18,26,27]
**GroEL–TLR** **Activation**	GroEL can be recognized by Toll-like receptors TLR4 and TLR2 found on epithelial and immune cells. This recognition activates the NF-κB- and MAPK-signaling pathways, leading to the production of pro-inflammatory cytokines, such as IL-8 and TNF-α. These cytokines may promote neutrophil recruitment and contribute to chronic mucosal inflammation. Additionally, GroEL’s interaction with TLR4 and TLR2 can activate NLRP3 inflammasomes, further driving the production of chemokines and cytokines.	[23,24]
**CagA/VacA** **Signaling**	Delivery of CagA and VacA via OMVs can result in changes to the cytoskeleton, disruption of tight junctions, vacuolization, and mitochondrial dysfunction. These alterations may promote cell proliferation and increase resistance to apoptosis.	[26,27]
**Exosomal Hsp60** **Release**	Oxidative, inflammatory, and bacterial stress increases Hsp60 levels in human gastric epithelial cells. Typically located in the mitochondria, Hsp60 moves to the plasma membrane under stress, is packaged into multivesicular bodies, and is secreted as exosomes, leading to elevated extracellular Hsp60 levels.	[10,28,29,30,31,32,33]
**Molecular** **Mimicry**	The release of Hsp60 from human exosomes in pre-neoplastic or dysplastic cells may facilitate immune recognition through molecular mimicry. This process could potentially lead to autoimmune reactions involving T cells or antibodies against gastric cells, which might enhance immune surveillance but could also contribute to tissue damage and carcinogenesis.	[25,34,35,36,37]
**Chronic** **Inflammatory** **Loop**	The production of OMVs by *H. pylori* is a crucial mechanism for its virulence and ability to evade the immune system. In response to this, the release of Hsp60 via human exosomes activates innate immune receptors such as TLR4 on macrophages and dendritic cells. This activation leads to an increased secretion of pro-inflammatory cytokines, including IL-6, IL-8, and TNF-α. This process sustains chronic inflammation typical of *H. pylori*-infected mucosa.	[14,15,16,23,24,30,33]
**MALT Lymphoid** **Induction**	The presence of GroEL in *H. pylori* OMVs contributes to a low-grade inflammatory state, marked by lymphoplasmacytic infiltration, neutrophil accumulation, and lymphoid follicle formation, which is associated with MALT lymphoma development.	[33]

## Data Availability

This review article does not report any new data. All data discussed in this work are available in the original publications cited in the reference list.
